# Expression and molecular consequences of inhibition of estrogen receptors in granulosa cells of bovine follicles

**DOI:** 10.1186/s13048-014-0096-0

**Published:** 2014-10-23

**Authors:** Monique Tomazele Rovani, Bernardo Garziera Gasperin, Gustavo Freitas Ilha, Rogério Ferreira, Rodrigo Camponogara Bohrer, Raj Duggavathi, Vilceu Bordignon, Paulo Bayard Dias Gonçalves

**Affiliations:** Laboratory of Biotechnology and Animal Reproduction – BioRep, Federal University of Santa Maria, Santa Maria, RS Brazil; Departament of Animal Pathology, Federal University of Pelotas, Capão do Leão, Brazil; Department of Animal Science, Santa Catarina State University, Chapecó, SC Brazil; Department of Animal Science, McGill University, Sainte Anne de Bellevue, QC Canada

**Keywords:** Estrogen receptors, Follicular deviation, Fulvestrant, Intrafollicular injection, Bovine

## Abstract

**Background:**

Estradiol (E2) receptors mediate E2 effects on cell proliferation and apoptosis under normal and pathological conditions. However, the mechanisms involved in E2 signaling are not completely understood. The objectives in this study were to evaluate the expression of estrogen receptors (ESRs) during follicular selection in cattle, and the effect of intrafollicular injection of fulvestrant (an antagonist of ESRs) on follicular development and transcript abundance in granulosa cells.

**Methods:**

Granulosa cells were obtained from the two largest follicles around follicular deviation, after FSH treatment and after intrafollicular injection of fulvestrant. Ovarian follicular dynamics monitored by ultrasonography and quantitative real time PCR were used to validate the *in vivo* model and investigate the effects of FSH supplementation or ESR blockade on mRNA expression of estradiol-related genes.

**Results:**

*ESR1* and *ESR2* were expressed in granulosa cells of both dominant (F1) and subordinate (F2) follicles, but their transcripts levels were higher in F1 than F2 after follicular deviation. FSH treatment maintained mRNA levels of both *ESR1* and *ESR2* in F2 follicles at similar levels observed in F1 follicles. Intrafollicular injection of 100 μM fulvestrant inhibited follicular growth and decreased *CYP19A1* mRNA levels. Transcript levels for both *ESR1* and *ESR2* were not affected by fulvestrant injection. Analyses of FSH-regulated genes revealed that ESRs inhibition in the dominant follicle decreased the transcript levels of the *GJA1* but not those of *PRKAR2B*, *MRO* or *LRP11* genes.

**Conclusions:**

Our findings indicate that: both *ESR1* and *ESR2* are regulated during follicular deviation and dominance in cattle and in response to FSH treatment, and ESRs are required for normal gene expression and development of the dominant follicle. Furthermore, we have validated an *in vivo* model to study estrogen signaling during follicular development that allows paracrine signaling between different follicular cells in a physiological endocrine environment.

## Background

Follicular deviation is characterized by the selection of one follicle while the other follicles become atretic. Dominant follicles (F1) have greater concentrations of estradiol (E2) in follicular fluid when compared to subordinate follicles (F2) [1,2]. It has been shown that E2 protects granulosa cells from apoptosis, promoting cell cycle progression in healthy follicles [3], whereas subordinate follicles lose their ability to produce E2 and undergo atresia [4]. Besides its pivotal role during normal follicle development, E2 signaling also regulates ovarian cancer cell proliferation and apoptosis [5], being ESRs important prognostic biomarkers for ovarian cancer [6].

It is well established that E2 signaling is mediated by the intracellular receptors estrogen receptor 1 (ESR1) and estrogen receptor 2 (ESR2), which are members of the nuclear receptor superfamily [7]. In mouse ovaries, ESR1 is mainly expressed in interstitial cells, whereas ESR2 is localized in granulosa cells of growing follicles [8]. In mice, females lacking *Esr1* gene are infertile and non-receptive to males, which indicates defective estrogen response in the central nervous system [9]. In order to circumvent the lack of ESR1-mediated action in the hypothalamic-pituitary axis, Couse et al. [10] administrated exogenous gonadotropins to *Esr1* knockout mice and confirmed that ESR1 is required for ovulation. On the other hand, *Esr2* knockout mice have lower number of growing follicles and reduced litter size compared to wild-type females [11].

Differentiation of granulosa cells in response to FSH is enhanced by estrogen [12,13]. Using *in vitro* knockout approaches, it was observed that ESR2 mediates estrogen actions. Indeed, ESRs were shown to be essential for differentiation of mouse granulosa cells in response to FSH, and a critical factor for expression of LH receptor (*LHCGR*) but not for FSH receptor (*FSHR*) [14,15]. It was also demonstrated that *ESR2* deletion impairs the cAMP pathway response to FSH, changing the pattern of global gene expression and attenuating the expression of various FSH-regulated genes [15]. In cattle, it was shown that *ESR2* mRNA expression is up regulated in fully differentiated follicles compared to subordinate follicles between days 2 and 3.5 of the estrous cycle [16]. However, the expression pattern of ESRs before, during and after follicle deviation has not been demonstrated. Moreover, the consequences of pharmacologic inhibition of ESRs during bovine follicular growth have not been investigated.

Intrafollicular injection in live animals represents an invaluable tool to investigate the physiological roles of ESRs during folliculogenesis. Indeed, the possibility of performing follicular manipulations *in vivo* while maintaining the complex follicular ultrastructure and cellular interactions circumvents the limitations of the *in vitro* models. Fulvestrant (ICI 182,780) is an antiestrogen that competes with E2 for binding to ESRs with no agonist activity [17]. Fulvestrant binds to ESRs and prevents their dimerization. The formed fulvestrant-ESR complexes are not translocated into the nucleus thereby culminating in the degradation of the complex [18].

In this study, we have used cattle as an *in vivo* model to: a) investigate the expression pattern of ESRs in the two largest follicles collected before, at the expected time-point, and after follicular deviation; b) evaluate the effect of FSH on ESRs expression; and c) determine the effects of ESRs inhibition on follicular development, and expression of ESRs and FSH-regulated genes in granulosa cells of developing follicles.

## Methods

### Animals

All procedures were approved by the Institutional Committee for Ethics in Animal Experiments at the Federal University of Santa Maria, RS, Brazil. Adult cyclic *Bos taurus taurus* beef cows were used in this study.

### Estrus synchronization and follicular growth monitoring

Cows used in experiments 1 and 2 (detailed below) were synchronized with two doses of a prostaglandin F2α (PGF2α) analogue (cloprostenol, 250 μg; Schering-Plough Animal Health, Brazil) given intramuscularly (i.m.) 11 days apart. Animals observed in estrus within 3–5 days after the second PGF2α administration were included in the experiments.

Cows used in experiment 3 were treated with a progesterone releasing intravaginal device (1 g progesterone, DIB – Intervet Schering Plough, Brazil), an im injection of 2 mg estradiol benzoate (Genix, Anápolis, Brazil) to induce follicular regression and emergence of a new follicular wave, and two (12 h apart) im injections of PGF2α. Four days later, the progesterone device was removed and ovaries were monitored daily for at least 3 days before treatment to ensure that new follicles were growing and persistent follicles were not present in the ovaries. Only cows without a corpus luteum in an ultrasound exam were included in the study to avoid progesterone inhibitory effects during the final stage of follicular growth and ovulation.

In all experiments, ovaries were examined once a day by transrectal ultrasonography, using an 8 MHz linear-array transducer (Aquila Vet scanner, Pie Medical, Netherlands) and all follicles larger than 5 mm were drawn using 3 to 5 virtual slices of the ovary allowing a three-dimensional localization of follicles and monitoring individual follicles during follicular wave [19].

### Ovary collection and isolation of granulosa cells

Cows were ovariectomized by colpotomy under caudal epidural anesthesia [20]. Ovaries were washed with saline and granulosa cells were harvested from follicles through repeated flushing with PBS. Cell samples were immediately stored in liquid nitrogen for further analyses.

### RNA extraction, reverse transcription and real-time PCR

Total RNA was purified from granulosa cells using silica-based protocol (Qiagen, Mississauga, ON, Canada) according to the manufacturer’s instructions. Quantitation and estimation of RNA purity was performed using a NanoDrop spectrophotometer (Thermo Scientific - Waltham, USA; Abs 260/280 nm ratio). Ratios above 1.8 were considered pure, and samples below this threshold were discarded. Complementary DNA was synthesized from 500 ng RNA, which was first treated with 0.1 U DNase, Amplification Grade (Life Technologies, Burlington, ON) for 5 min at 37°C. After DNase inactivation at 65°C for 10 min, samples were incubated in a final volume of 20 μl with iScript cDNA Synthesis Kit (BioRad, Hercules, CA). Complementary DNA synthesis was performed in three steps: 25°C – 5 min, 42°C – 30 min and 85°C – 5 min.

To test cross-contamination with theca cells, quantitative PCR detection of Cytochrome P450, family 17, subfamily A, polypeptide 1 mRNA (*CYP17A1*; NM_174304.2; F: CCATCAGAGAAGTGCTCCGAAT; R: GCCAATGCTGGAGTCAATGA) was performed in granulosa cells. Samples were considered free of contamination if *CYP17A1* was not amplified within 30 PCR cycles. Quantitative polymerase chain reactions (qPCR) were conducted in a CFX384 thermocycler (BioRad) using iQ SYBR Green Supermix (BioRad) and bovine-specific primers (Table 1) taken from the literature or designed using the Primer Express Software (Applied Biosystems). Standard two-step qPCR was performed with initial denaturation at 95°C for 5 min followed by 40 cycles of denaturation at 95°C for 15 sec and annealing/extension at 58°C for 30 sec. Melting-curve analyses were performed to verify product identity.

**Table 1:** **List of primers used in the qPCR reactions**

**Gene name**	**Primer sequence (5′ to 3′)**	**Reference or accession no.**
*CYP19A1*	F: GTGTCCGAAGTTGTGCCTATT	[21]
	R: GGAACCTGCAGTGGGAAATGA	
*ESR1*	F: CCAACCAGTGCACGATTGAT	NM_001001443.1
	R: TTCCGTATTCCGCCTTTCAT	
*ESR2*	F: CAGCCGTCAGTTCTGTATGCA	NM_174051.3
	R: TCCTTTTCAATGTCTCCCTGTTC	
*FSHR*	F: AGCCCCTTGTCACAACTCTATGTC	[21]
	R: GTTCCTCACCGTGAGGTAGATGT	
*GJA1*	F: GTCTTCGAGGTGGCCTTCTTG	NM_174068.2
	R: AGTCCACCTGATGTGGGCAG	
*LHCGR*	F: GCACAGCAAGGAGACCAAATAA	NM_174381.1
	R: TTGGGTAAGCAGAAACCATAGTCA	
*LRP11*	F: CCAGAAAGTCGCATTGATCTTG	NM_001206831.1
	R: TGTTCCCCTCCTCCTCGATT	
*MRO*	F: CCCACTTACAGGACAGGAATCC	NM_001034552.1
	R: TGGAAGCTGTAGTCCTTGCTTTG	
*PRKAR2B*	F: GGGCATTCAACGCTCCAGTA	NM_174649.2
	R: CTGGATTCAGCATCATCTTCTTCTT	

F, Forward primers; R, Reverse primers.

To optimize the qPCR assay, serial dilutions of cDNA templates were used to generate a standard curve. The standard curve was constructed by plotting the log of the starting quantity of the dilution factor against the Ct value obtained during amplification of each dilution. Reactions with a coefficient of determination (R2) higher than 0.98 and efficiency between 95 to 105% were considered optimized. The relative standard curve method was used to assess the amount of a particular transcript in each sample [22]. Samples were run in duplicate and results are expressed relative to *GAPDH* (NM_001034034.2; F: ACCCAGAAGACTGTGGATGG; R: CAACAGACACGTTGGGAGTG), *cyclophilin B* (NM_174152.2; F: GGTCATCGGTCTCTTTGGAA; R: TCCTTGATCACACGATGGAA), *RPL19* (NM_001040516.1; F: GCCAACTCCCGTCAGCAGA; R: TGGCTGTACCCTTCCGCTT) and/or *RPLP0* (NM_001012682.1; F: GGCGACCTGGAAGTCCAACT; R: CCATCAGCACCACAGCCTTC) or the average Ct values for these genes as internal controls. The selection of the internal control genes was based on the Ct variance (as reflected by the standard deviation) among groups in each experiment.

### Experiment 1: Estrogen receptors expression in granulosa cells around the period of follicle deviation

Thirty-two cows were synchronized, of which the fifteen cows that were detected in estrus 3 to 5 days after the second PGF2α administration were ovariectomized at specific stages of the first follicular wave. The day of follicular emergence was designated as day-0 of the wave and was retrospectively identified as the last day on which the dominant follicle was 4 to 5 mm in diameter [23]. Separate groups of cows were randomly assigned for ovariectomy on days-2 (n = 4), 3 (n = 4) or 4 (n = 7) of the follicular wave to recover the two largest follicles from each cow. This approach allowed us to investigate transcript abundance of *ESRs* and related genes when the size of the largest and second largest follicle did not have a significant difference (day-2 of the follicular wave), had slight difference (day-3) or marked difference (day-4). These time-points correspond to before, during and after the dominant follicle selection, respectively.

### Experiment 2: Estrogen receptor expression after FSH treatment

This experiment was conducted to compare mRNA levels of *ESR* genes between the two largest follicles collected from FSH (n = 3) and saline (n = 4) treated cows. FSH treated cows received two doses of 30 mg FSH (Folltropin-V, Bioniche Animal Health, Ontario, Canada) on the second day of the estrous cycle followed by two doses of 20 mg on the third day. Control cows were injected at the same time with saline. Ovaries were collected 12 hours after the last FSH/saline treatment and granulosa cells were recovered as described above.

### Experiment 3: Effect of intrafollicular administration of an estrogen receptor inhibitor on follicular development and gene expression in granulosa cells

To determine the effective dose of the estrogen receptor inhibitor, fulvestrant (Sigma–Aldrich, Brazil), nine adult cyclic cows were synchronized as detailed above and their ovaries were monitored by transrectal ultrasonography. When the largest follicle of the growing cohort reached a diameter between 7 to 8 mm, which represents the size when the future dominant follicle is reliably identifiable [24,25], it was injected with 1, 10 or 100 μM (n =3/group) fulvestrant. Intrafollicular injection and adjustment of fulvestrant amount to be injected according to follicular size were performed as previously described [26]. The development of the injected follicles was monitored by daily ultrasound examination for three days after treatment.

Based on the inhibition of follicular growth (see the Results), the highest concentration of fulvestrant (100 μM) was chosen to evaluate the effect of ESRs inhibition on gene expression in granulosa cells. Six cows were synchronized and their future dominant follicle was injected intrafollicularly with fulvestrant or saline (n = 3 per group). Cows were ovariectomized at 12 h after intrafollicular injection to harvest granulosa cells.

### Statistical analyses

Variation in transcript levels was analysed by ANOVA and multiple comparisons between days or groups were performed by LSMeans Student’s *t* test using the JMP Software. Continuous data were tested for normal distribution using Shapiro–Wilk test and normalized when necessary. The effect of fulvestrant on follicular development was performed as repeated measures data using the MIXED procedure with a repeated measure statement using the SAS Software package (SAS Institute, Inc., Cary, NC, USA). Main effects of treatment group, day, and their interaction were determined. Differences between follicular sizes at a specific time point were compared between groups using estimates. Differences between the two largest follicles were accessed by paired Student’s *t*-test using cow as subject. Results are presented as means ± S.E.M. P ≤0.05 was considered statistically significant.

## Results

### Expression of ESRs during follicular selection and dominance

The respective diameters of the largest (F1) and the second largest (F2) follicles collected before (day-2), during (day-3) and after (day-4) deviation were 7.3 ± 0.2 mm and 6.4 ± 0.1 mm (P >0.05), 8.1 ± 0.2 mm and 6.5 ± 0.4 mm (P >0.05), and 9.5 ± 0.2 and 6.8 ± 0.1 (P <0.0001). Using the same experimental model, we have previously shown that estradiol concentrations increased in the follicular fluid of F1 (dominant) follicles from day 2 to 4, but decreased in F2 (subordinate) follicles on days 3 and 4 [24].

**Figure 1:**
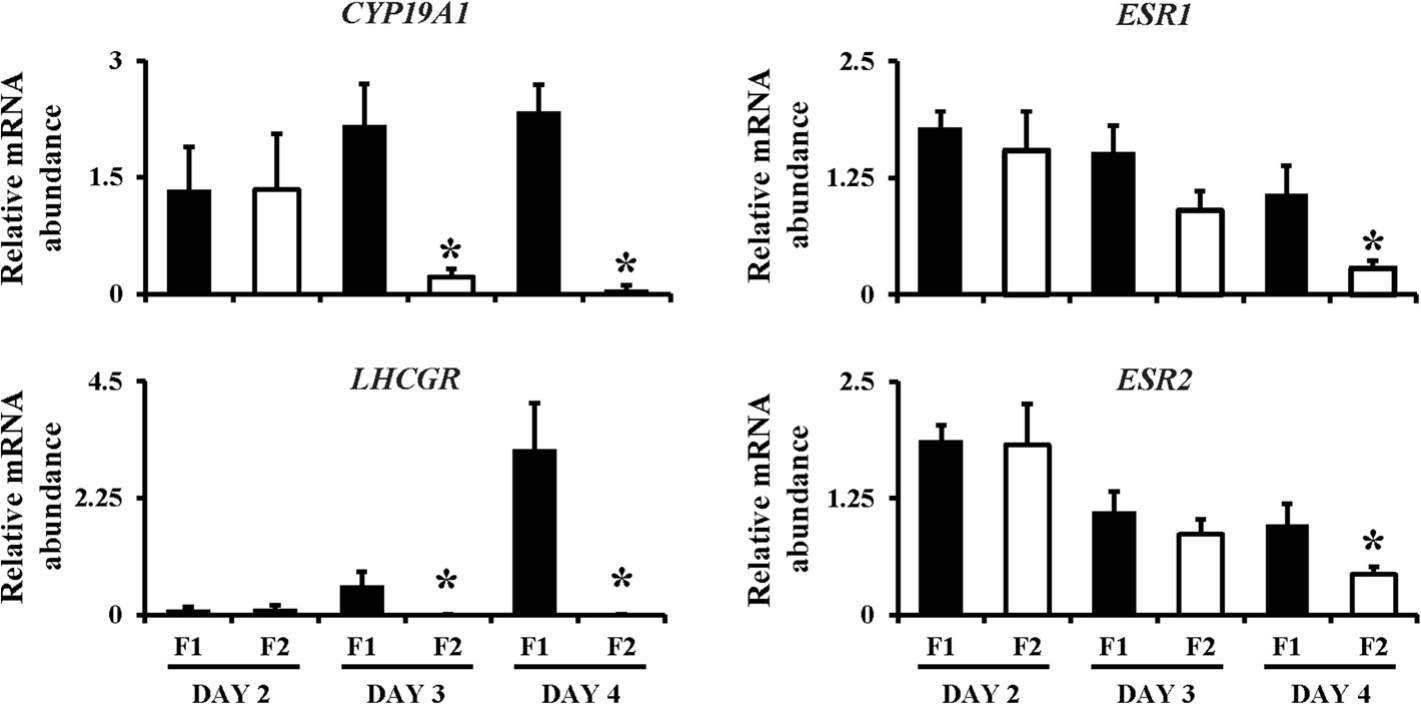
**Relative mRNA abundance in bovine granulosa cells during follicular deviation.** The two largest follicles from each cow were collected from the ovaries of 15 cows on days – 2 (n =4), 3 (n =4) or 4 (n =7) of the first follicular wave. Abundance of *CYP19A1*, *LHCGR*, *ESR1* and *ESR2* genes are expressed as mean ± SEM. * indicates statistical difference (P ≤0.05) between the largest (F1) and second largest (F2) follicles.

In order to validate the *in vivo* experimental models, we first assessed mRNA levels of Cytochrome P450, family 19, subfamily A, polypeptide 1 (*CYP19A1*) and LH receptor (*LHCGR*) genes in granulosa cells from the largest and second largest follicles on days-2 (n = 4), 3 (n = 4) or 4 (n = 7) of the follicular wave. Subordinate follicles expressed low levels of *CYP19A1* and *LHCGR* (Figure 1) during (day-3) and after (day-4) the expected time of follicular deviation. The relative mRNA abundance of *ESR1* and *ESR2* in granulosa cells was then compared between the largest (F1) and second largest (F2) follicles (Figure 1). While mRNA levels of ESRs were similar between F1 and F2 follicles before (day-2) and at (day-3) the expected time of follicular deviation, both *ESR1* and *ESR2* transcripts were higher (P <0.05) in F1 than F2 follicles after deviation (day-4).

### Effect of FSH treatment on ESR expression

**Figure 2:**
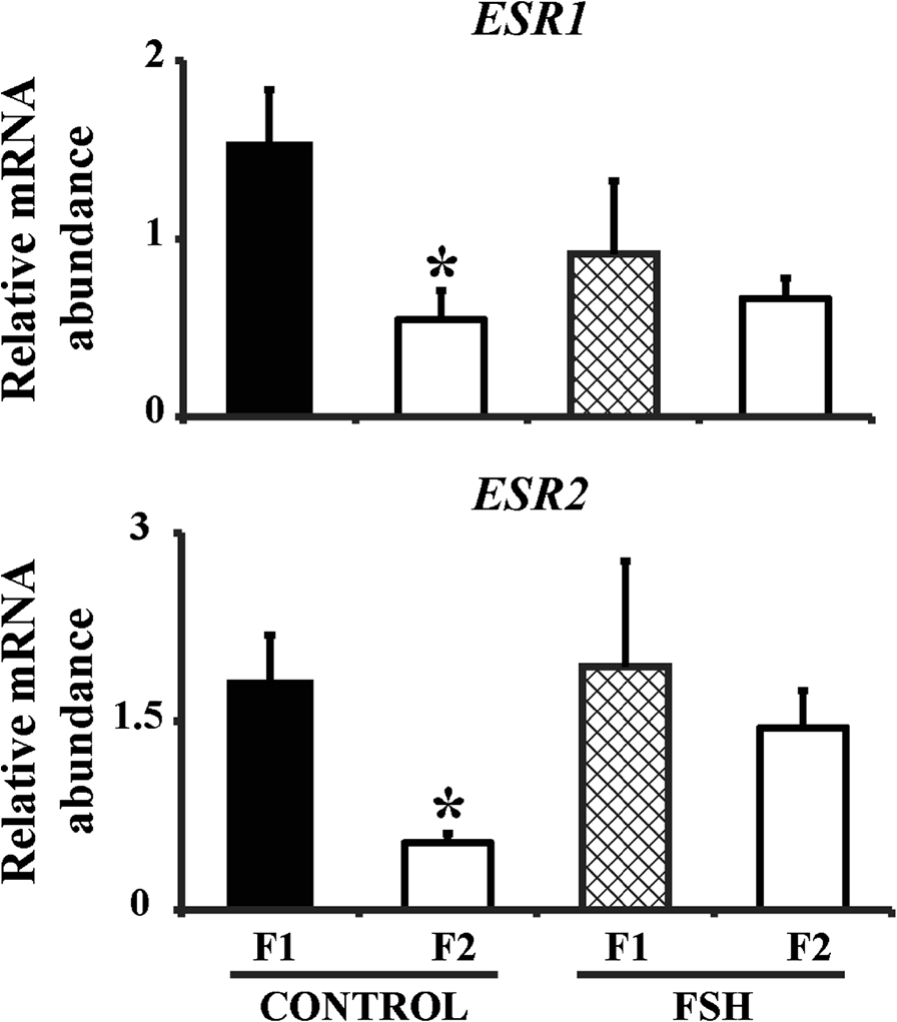
**Relative mRNA abundance in granulosa cells of the two largest follicles in saline or FSH-treated cows.** Cows were treated twice a day (12 h apart) with FSH (30, 30, 20 and 20 mg) or saline (control) starting on day 2 after ovulation. Granulosa cells were collected from the two largest follicles 12 h after the last administration of FSH (n = 4 pairs) or saline (n = 3 pairs). Abundance of *ESR1* and *ESR2* are expressed as mean ± SEM. * indicates statistical difference (P ≤0.05) between largest and second largest follicles.

Based on the findings of the first study we evaluated whether FSH treatment would maintain normal expression of ESRs in the second largest follicles. Similarly to the first experiment, we confirmed that mRNA levels of ESRs were higher (P <0.05) in F1 than F2 follicles after deviation (Figure 2). Yet, there was no difference (P > 0.05) in either *ESR1* or *ESR2* mRNA levels between F1 and F2 follicles collected from FSH-treated animals (Figure 2). CYP19A1 mRNA abundance did not differ (p > 0.05) between F1 (0.6 ± 0.13) and F2 (0.33 ± 0.11) follicles of FSH-treated cows, but was significantly higher (p < 0.05) in F1 (1 ± 0.18) compared to F2 (0 ± 0) follicles from control cows.

### Effect of intrafollicular inhibition of ESRs on follicular development and ESRs expression

**Figure 3:**
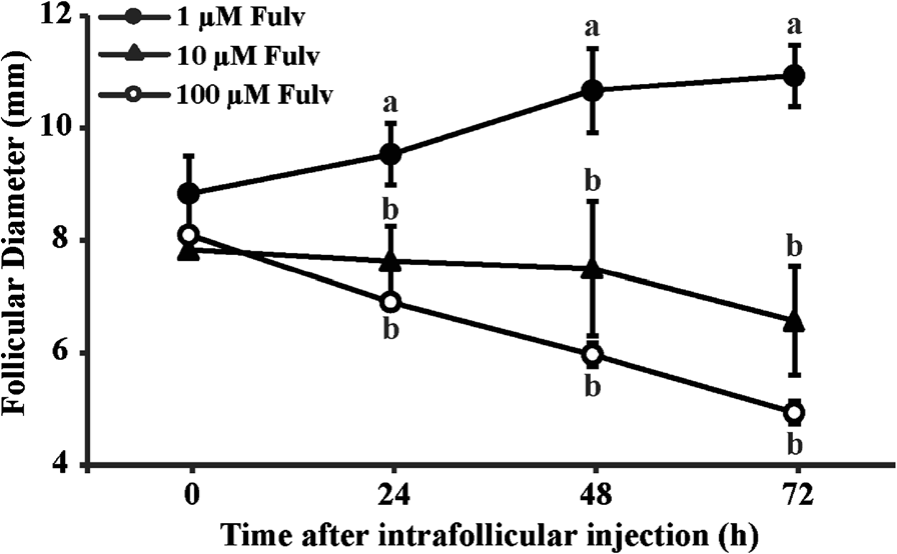
**Effect of intrafollicular injection of an estrogen-receptor antagonist (fulvestrant) on follicular growth.** A new follicular wave was induced (detailed in Methods) and 1, 10 or 100 μM fulvestrant (n = 3/group) was intrafollicularly injected when the largest follicle reached a diameter between 7 to 8 mm. Follicular diameters were monitored by daily ultrasound examinations until 72 h after intrafollicular treatment. Different letters indicate significant differences (P ≤0.05) between treatments within a time.

Our next objective was to evaluate the consequences of inhibiting ESRs in growing follicles around the time of follicular deviation. We first monitored follicular growth in response to intrafollicular injection of 1, 10 or 100 μM fulvestrant in follicles having an average diameter of 8.8 ± 0.6, 7.8 ± 0.1 and 8.1 ± 0 mm (P >0.05), respectively. While follicular development was inhibited by the higher concentrations (10 and 100 μM) of fulvestrant (Figure 3; P ≤0.01), follicles injected with 1 μM continued developing. This confirmed that the inhibition of follicular growth was specifically due to the higher concentration of fulvestrant rather than as a consequence of the intrafollicular injection procedure.

**Figure 4:**
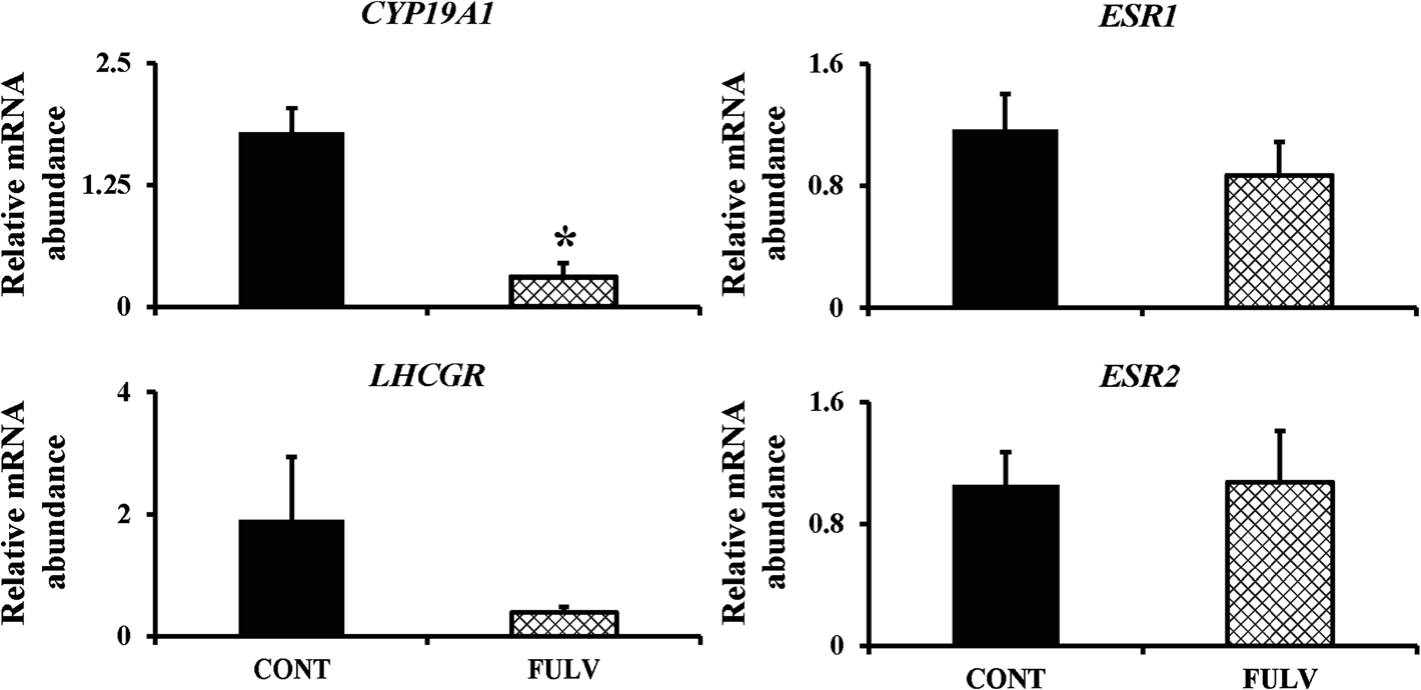
**Relative mRNA abundance in granulosa cells of the largest follicle after intrafollicular injection of fulvestrant.** A new follicular wave was induced (detailed in Methods) and 100 μM fulvestrant or saline was intrafollicularly injected when the largest follicle reached a diameter between 7 to 8 mm. Granulosa cells were recovered from saline (n = 3) and fulvestrant (n = 3) treated follicles at 12 h after intrafollicular injection. Abundance of *CYP19A1*, *LHCGR*, *ESR1* and *ESR2* genes are expressed as mean ± SEM. * indicates statistical difference (P ≤0.05) between groups.

As expected, intrafollicular inhibition of ESRs with 100 μM fulvestrant resulted in decreased abundance (P ≤0.05) of mRNA encoding *CYP19A1* (Figure 4). However, mRNA levels of *LHCGR*, *ESR1* and *ESR2* were not different between control and fulvestrant-injected follicles (Figure 4).

### Effect of ESRs inhibition on the expression of FSH-regulated genes in granulosa cells

**Figure 5:**
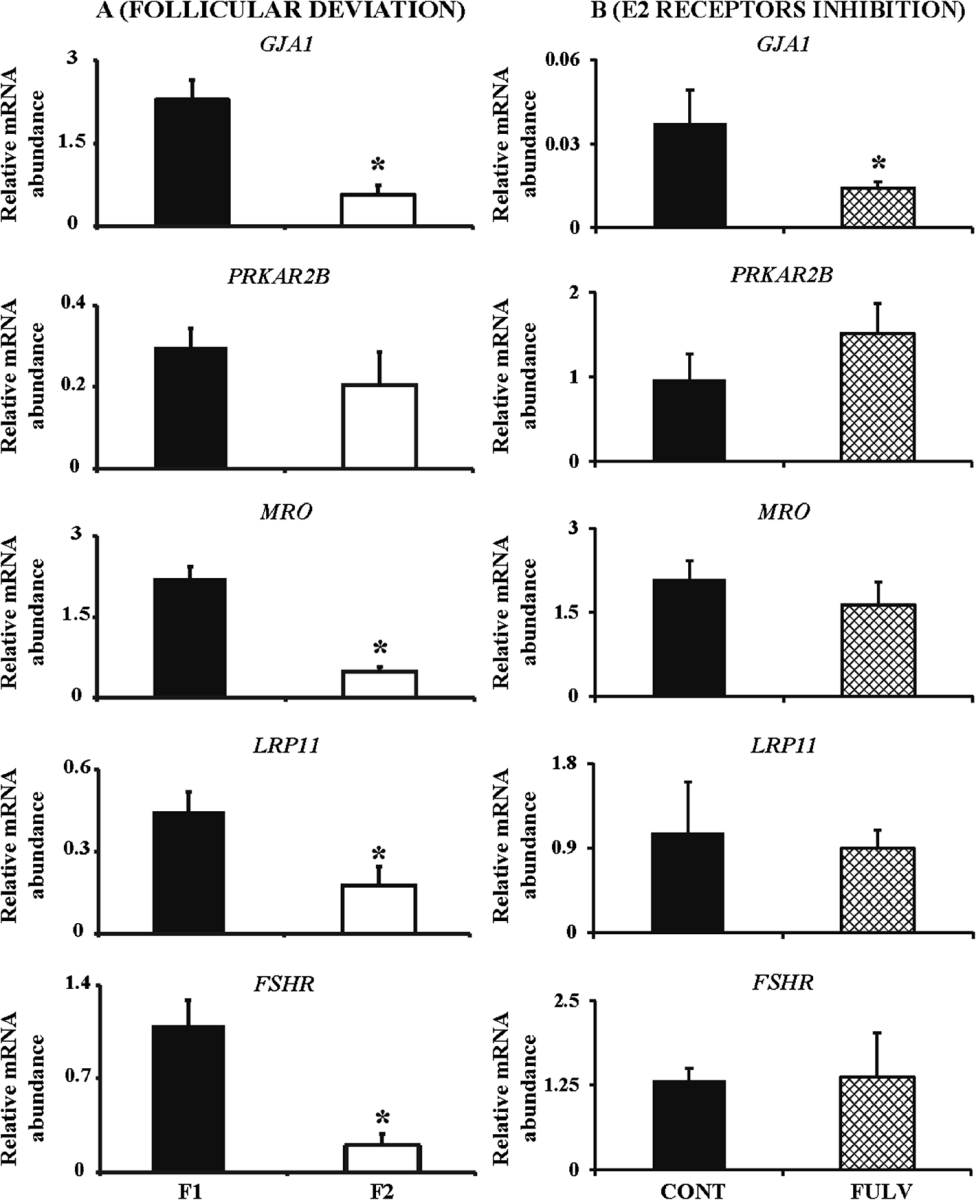
**Relative mRNA abundance in granulosa cells after follicular deviation (A) and after fulvestrant treatment (B).** Abundance of *GJA1*, *PRKAR2B*, *MRO*, *LRP11* and *FSHR* genes are expressed as mean ± SEM. In **A**, asterisk (*) indicates statistical difference (P ≤0.05) between largest (F1) and second largest (F2) follicles after follicular deviation. In **B**, asterisk (*) indicates statistical difference (P ≤0.05) between groups: intrafollicular injection of saline or fulvestrant.

Our final objective was to evaluate the effect of intrafollicular administration of 100 μM fulvestrant on granulosa cell gene expression. The diameters of F1 and F2 follicles used in this experiment were 9.5 ± 1.3 mm and 6.1 ± 0.6 mm (P <0.05) in the control group, and 8.6 ± 0.9 mm and 7.5 ± 0.7 mm (P >0.05) in the FSH-treated group. We focused on FSH-regulated genes such as Gap junction protein alpha 1 (*GJA1*), Maestro (*MRO*), Low density lipoprotein receptor-related protein 11 (*LRP11*), *FSHR* and Protein kinase, cAMP-dependent, regulatory, type II, beta (*PRKAR2B*), as these were reported to be downregulated in granulosa cells of *Esr2* null mice [15]. We first examined if these genes are indeed differentially regulated in dominant and subordinate follicles using granulosa cells of F1 and F2 collected on day 4 of the follicular wave (Experiment 1). Relative mRNA levels of *GJA1*, *MRO*, *LRP11*, *FSHR*, but not *PRKAR2B*, were higher (P ≤0.05) in granulosa cells of F1 than F2 follicles (Figure 5A). However, in granulosa cells of fulvestrant-treated follicles only *GJA1* mRNA was lower (P ≤0.05) compared to granulosa cells of control follicles (Figure 5B). Fulvestrant treatment did not affect (P >0.05) the abundance of mRNA encoding *PRKAR2B*, *MRO*, *LRP11* and *FSHR* relative to granulosa cells of control follicles (Figure 5B).

## Discussion

In this study, cattle were used as an *in vivo* model to investigate regulation of *ESR 1* and *2* during follicular deviation in a monovular species, and the effects of intrafollicular inhibition of ESRs on follicular growth and gene expression. We observed that: expression of *ESR1* and *2* was higher in granulosa cells of the largest compared to second largest follicle after deviation; FSH maintained expression of both ESRs in the second largest follicles beyond the follicular deviation; inhibition of ESRs abrogated follicular growth without decreasing their transcript levels and; FSH-regulated genes respond differently to intrafollicular inhibition of ESRs in growing follicles.

Studies with mice have established that ESR2 is the receptor responsible for mediating estrogen actions in granulosa cells [8,14,15]. However, ESR1 has been proposed to be the main receptor involved in follicular development in cattle [27]. This suggests that regulation of ESRs may differ between monovulatory and polyovulatory species. In this study, we have confirmed that both *ESR1* and *ESR2* are expressed in granulosa cells during follicular selection in cattle. While the expression of *ESR1* and *ESR2* was significantly decreased in granulosa cells of the subordinate follicle after deviation, both ESRs were constitutively expressed in the selected dominant follicle. It is therefore possible that both receptors are required for the continued development of the dominant follicle during and after follicular deviation in cattle. Our primary objective in this study was to characterize the expression of ESRs before, during and after follicle deviation. Although expected to be regulated before follicle deviation, we observed that both ESR1 and ESR2 were regulated after deviation. This suggests that ESRs are not involved in follicular selection, but they are necessary to maintain follicular development of the selected follicles.

Although previous studies in rats have shown that hypophysectomised females express ESRs in granulosa cells in response to FSH [28], the effect of FSH treatment on the expression of ESRs during follicular growth has not been thoroughly investigated in cattle. Herein, we found that FSH maintained the expression of both *ESR1* and *ESR2* in the second largest follicle at similar levels observed in the largest follicle, while mRNA levels for both ESRs were reduced in the second largest follicle of saline treated cows. This suggests that similar to rodents, FSH promotes the expression of both ESRs in granulosa cells during follicular growth and selection in cattle.

To further investigate the roles of ESRs during follicular growth, we performed *in vivo* intrafollicular administration of the ESRs antagonist fulvestrant in cows. Fulvestrant is known to disrupt the dimerization and accelerate the degradation of ESRs [29,30]. We first confirmed that fulvestrant injection suppresses follicular growth in a dose depend manner, which, in addition to validate our *in vivo* model, indicated that ESRs are required for continued development of the dominant follicle after deviation in cattle. Intrafollicular injection in cattle is a well-established technique. We and others have previously shown that this procedure did not affect follicle growth and ovulation [25,26,31,32]. Indeed, the fact that follicles injected with the lower concentration of fulvestrant (1 μM) continued to grow indicates that the intrafollicular injection procedure did not interfere with follicular development. Moreover, our data clearly show that only the higher concentrations of fulvestrant (10 or 100 μM) were able to block follicular growth and reduce CYP19A1 mRNA levels in granulosa cells. Estradiol concentrations were not determined in this study because we have previously reported that estradiol tended to be lower in the follicular fluid of fulvestrant-treated follicles compared to control follicles [33]. The inhibition of estrogen binding to its receptors by fulvestrant injection decreased the expression of *CYP19A1*, the enzyme responsible for androgen aromatization to estrogen, suggesting that estrogen regulates its own synthesis [34,35]. This is supported by our results from the follicular deviation model, where *CYP19A1* mRNA levels were lower in subordinate follicles collected on day- 3 and 4, which are known to have low estrogen levels [24,33]. Moreover, estrogen treatment has been shown to increase ESRs expression in granulosa cells of hypophysectomised rats [28]. On the other hand, we observed that transcripts levels of *ESR1* and *ESR2* were not affected by fulvestrant treatment. The aforementioned results validate fulvestrant intrafollicular injection as a valuable model to study estradiol signaling in granulosa cells. However, a model to study the specific functions of ESR1 and ESR2 still needs to be validated.

Using knockout mice, Deroo et al. [15] identified FSH-regulated genes that require *Esr2* for normal expression. Indeed, granulosa cells lacking *Esr2* had lower transcript levels of *Comp*, *Mro* and *Lrp11* genes after gonadotropin stimulation, whereas *Prkar2b* expression was not affected. In the present study, we observed no differences in transcript abundance of *PRKAR2B*, *MRO* or *LRP11* genes in response to inhibition of ESR signaling. This suggests that pharmacological inhibition of ESRs was not sufficient to downregulate *MRO* and *LRP11* in monovulatory compared to polyovulatory species. It is still possible that genetic deletion of ESRs may result in phenotype similar to rodents. On the other hand, we observed that follicles treated with fulvestrant had significantly decreased mRNA levels of *GJA1* compared to control follicles. The GJA1 provides the communication among granulosa cells via gap junction channels, and it was shown to be highly expressed in granulosa cells of rat follicles [36]. It has been well established that GJA1 is the most important connexin that makes a significant contribution to intercellular coupling in mouse granulosa cells and *Gja1* null mice exhibit aberrant follicular growth [37]. It has also been reported that GJA1 mRNA and protein decrease during follicular atresia induced by E2 withdrawal in rodents [38]. Our findings with intrafollicular injection of fulvestrant demonstrate that the ESR signalling is necessary for *GJA1* expression in granulosa cells of growing follicles of monovulatory species. Further, these observations indicate that inhibition of ESRs abrogates follicular growth at least in part through deregulated intercellular communication among granulosa cells. Taken together, our results indicate that the *in vivo* model used in this study represents an important asset to investigate steroid hormones signaling mechanisms in the ovary, which is needed for advancing our understanding of both physiological and pathological conditions [6].

## Conclusions

Using an *in vivo* model of a monovulatory species, we have shown that both *ESR1* and *ESR2* are regulated in granulosa cells during follicular deviation and dominance, and in response to FSH treatment. Moreover, by intrafollicular injection of an antagonist, we confirm that ESRs are required for the normal development of the dominant follicle in cattle. Finally, we propose that intrafollicular injection in cattle is a suitable *in vivo* model to study estrogen signaling during follicular deviation and dominance in monovulatory species.
